# CLERK is a novel receptor kinase required for sensing of root-active CLE peptides in *Arabidopsis*

**DOI:** 10.1242/dev.162354

**Published:** 2018-05-22

**Authors:** Pauline Anne, Amelia Amiguet-Vercher, Benjamin Brandt, Lothar Kalmbach, Niko Geldner, Michael Hothorn, Christian S. Hardtke

**Affiliations:** 1 Department of Plant Molecular Biology, University of Lausanne, Biophore Building, CH-1015 Lausanne, Switzerland; 2 Structural Plant Biology Laboratory, Department of Botany and Plant Biology, University of Geneva, CH-1211 Geneva, Switzerland

**Keywords:** Receptor kinase, CLE peptide, Protophloem, Root, BAM3, *Arabidopsis*

## Abstract

CLAVATA3/EMBRYO SURROUNDING REGION (CLE) peptides are secreted endogenous plant ligands that are sensed by receptor kinases (RKs) to convey environmental and developmental inputs. Typically, this involves an RK with narrow ligand specificity that signals together with a more promiscuous co-receptor. For most CLEs, biologically relevant (co-)receptors are unknown. The dimer of the receptor-like protein CLAVATA 2 (CLV2) and the pseudokinase CORYNE (CRN) conditions perception of so-called root-active CLE peptides, the exogenous application of which suppresses root growth by preventing protophloem formation in the meristem. *clv2* as well as *crn* null mutants are resistant to root-active CLE peptides, possibly because CLV2-CRN promotes expression of their cognate receptors. Here, we have identified the *CLE-RESISTANT RECEPTOR KINASE* (*CLERK*) gene, which is required for full sensing of root-active CLE peptides in early developing protophloem. CLERK protein can be replaced by its close homologs, SENESCENCE-ASSOCIATED RECEPTOR-LIKE KINASE (SARK) and NSP-INTERACTING KINASE 1 (NIK1). Yet neither CLERK nor NIK1 ectodomains interact biochemically with described CLE receptor ectodomains. Consistently, *CLERK* also acts genetically independently of *CLV2-CRN*. We, thus, have discovered a novel hub for redundant CLE sensing in the root.

## Abstract (precis)

**Highlighted Article:** The intricate interplay between CLE-sensing pathways along the spatiotemporal gradient of protophloem formation involves the novel *CLE-RESISTANT RECEPTOR KINASE* gene.

## INTRODUCTION

The endogenous CLAVATA3/EMBRYO SURROUNDING REGION (CLE) peptides of plants are secreted as propeptides and processed to yield bioactive 12-14 amino acid long ligands (Cock and McCormick, 2001; Ito et al., 2006; Kondo et al., 2006). The *Arabidopsis thaliana* genome contains 32 *CLE* genes, which encode 27 distinct CLE peptides (Goad et al., 2016; Ito et al., 2006; Strabala et al., 2006). Exogenous application of chemically synthesized CLE peptides at nanomolar concentrations frequently suppresses *Arabidopsis* root growth in tissue culture (Depuydt et al., 2013; Fiers et al., 2005; Hazak et al., 2017; Kinoshita et al., 2007; Miwa et al., 2008). With a few exceptions, the receptors and/or co-receptors for such root-active CLEs have not been identified. Generally, known CLE receptors fall into the category of receptor kinases (RKs) with extracellular leucine-rich repeat (LRR) domains. For example, the LRR-RK CLAVATA 1 (CLV1) directly binds the prototypical CLV3 peptide to regulate stem cell homeostasis in shoot meristems (Brand et al., 2000; Clark et al., 1995; Fletcher et al., 1999). PHLOEM INTERCALATED WITH XYLEM (PXY; also known as TDIF RECEPTOR) perceives the identical CLE41 and CLE44 peptides (a.k.a. TRACHEARY ELEMENT DIFFERENTIATION INHIBITORY FACTOR) to regulate vascular development in secondary growth (Etchells and Turner, 2010; Fisher and Turner, 2007; Hirakawa et al., 2008; Morita et al., 2016). In the root meristem, the LRR-RK BARELY ANY MERISTEM 3 (BAM3) is necessary for CLE45-triggered suppression of protophloem sieve element differentiation (Depuydt et al., 2013; Hazak et al., 2017; Kang and Hardtke, 2016). The protophloem is the first tissue to differentiate in the root meristem and represents the ultimate conduit of source-derived phloem sap into the meristem, which is a continuously growing sink organ. The two protophloem strands are each composed of a sieve element cell file and two neighboring companion cell files. Recently, it has been demonstrated that root-active CLE peptides prevent sieve element differentiation (Hazak et al., 2017). Therefore, efficient delivery of phloem sap into the meristem, and consequently root growth, is strongly inhibited (Hazak et al., 2017; Rodriguez-Villalon et al., 2015). Interestingly, only a few CLEs are expressed in the root meristem or the protophloem (Jun et al., 2010). Notable exceptions are CLE26 and CLE45, which are thought to act as autocrine signals in the protophloem differentiation process (Czyzewicz et al., 2015; Depuydt et al., 2013; Rodriguez-Villalon et al., 2014, 2015). Genetic analyses suggest that CLE45 and its cognate receptor BAM3 (Hazak et al., 2017) oppose the activity of positive regulators of protophloem sieve element differentiation, such as BREVIS RADIX (BRX) or OCTOPUS (OPS) (Depuydt et al., 2013; Rodriguez-Villalon et al., 2014, 2015). That is, second-site *bam3* null mutations can fully suppress the protophloem differentiation defects observed in *brx* or *ops* loss-of-function mutants (Depuydt et al., 2013; Rodriguez-Villalon et al., 2015). Thus, CLE45-BAM3 action is possibly required to keep developing protophloem cells in the dividing meristematic state, thereby preventing their premature transition to differentiation. However, because *bam3* null mutants do not display a root phenotype (Depuydt et al., 2013; Rodriguez-Villalon et al., 2014, 2015), this might involve redundant pathways, for example signaling by CLE26 (Czyzewicz et al., 2015; Rodriguez-Villalon et al., 2015), the receptor for which is unknown.

In numerous cases, ligand sensing by LRR-RKs requires interaction with co-receptor kinases (Brandt and Hothorn, 2016; Hazak and Hardtke, 2016; Katsir et al., 2011; Li and Tax, 2013). Notably, the SOMATIC EMBRYOGENESIS RECEPTOR KINASE (SERK) family of co-receptors, which are themselves LRR-RKs, have been implicated in various signal transduction pathways (Kemmerling et al., 2007; Meng et al., 2015; Santiago et al., 2016, 2013; Schwessinger and Rathjen, 2015; Torii, 2012). SERKs have also been implicated in PXY-mediated CLE41/44 signaling, although binding of PXY-CLE41/44 to SERKs is comparatively weak (Hazak et al., 2017; Santiago et al., 2016; Zhang et al., 2016a,b). Whether SERKs have a more-general role in CLE perception remains unclear, because single *serk* mutants are not resistant to root-active CLE peptides, and higher order *serk* mutants display pleiotropic phenotypes, including impaired root growth (Hazak et al., 2017).

An unusual component required for full-scale sensing of root-active CLE peptides is the dimer formed by the receptor-like protein (RLP) CLAVATA2 (CLV2) and the pseudokinase CORYNE (CRN) (Fiers et al., 2005; Hazak et al., 2017; Meng and Feldman, 2010; Miwa et al., 2008; Muller et al., 2008). Together, the extracellular LRRs and transmembrane domain of CLV2, and the transmembrane domain and intracellular pseudokinase domain of CRN, form a receptor-like complex (Brand et al., 2000; Jeong et al., 1999; Muller et al., 2008; Nimchuk et al., 2011). Recent results demonstrate that CLV2-CRN expression in the root protophloem is required for perception of root-active CLE peptides, and, at least in this context, CLV2-CRN appears to confer stable, efficient expression of the bona fide CLE45 receptor BAM3 (Hazak et al., 2017). This quantitative action of CLV2-CRN could explain why CLE resistance of *clv2* or *crn* mutants is not always fully penetrant. Alternatively, additional, and partially redundant CLE peptide perception pathways could exist. Here, we present the isolation of a previously uncharacterized LRR-RK, which is required for sensing of root-active CLE peptides in a genetically CLV2-CRN-independent fashion.

## RESULTS

### Identification of CLE26- and CLE45-responsive genes in the VISUAL assay

**Fig. 1.**
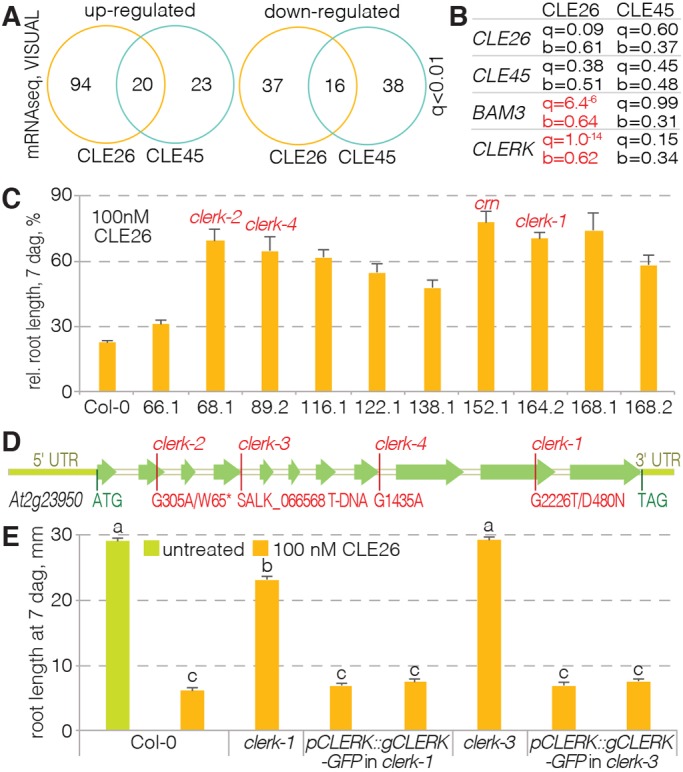
**Identification of the *CLERK* gene.** (A) Overview of the results from transcriptomic RNAseq analyses of CLE26 and CLE45 responses in the VISUAL assay for the genes with the most significant expression changes (q value<0.01). (B) Expression changes for selected genes upon CLE26 or CLE45 treatment in the VISUAL assay, with significance (q value) and fold change (b value) indicated. Significant changes are marked in red. b values represent mock divided by treatment. (C) Quantification of root growth in the presence of 100 nM CLE26 for 10 confirmed CLE26-resistant mutants. Causative mutated loci, as far as known, are indicated (red). All mutant values were statistically significantly different when compared with Col-0 wild type (ANOVA, *P*<0.001; *n*=8-21). (D) Schematic presentation of the UTR (light green) and intron-exon structure (in white and green, respectively) of the *CLERK* gene, with isolated mutant alleles indicated. (E) Complementation of CLE26-resistant root growth of *clerk* loss-of-function mutants by transgenic wild-type *CLERK* constructs (ANOVA, *P*<0.001; *n*=28-47). Statistically significant differences are indicated by letters (Tukey test, alpha=0.05). See Table S1 for full Tukey test results and ANOVA tables. Error bars represent s.e.m.

In an attempt to isolate novel components of CLE peptide signaling in *A. thaliana* root protophloem development, we embarked on a combinatorial approach of transcriptomic analysis and forward genetics. Experimental investigation of developing protophloem sieve elements is inherently difficult, because they represent only 40-50 cells inside the vascular cylinder of the root meristem, and some of our genes of interest are expressed only in a subset of these. Protophloem-specific transcriptomic responses are thus strongly diluted when root tips are assayed, and attempts to identify, e.g. protophloem-specific CLE45-responsive genes have so far been unsuccessful (Depuydt et al., 2013). However, in a novel trans-differentiation assay [vascular cell induction culture system using *Arabidopsis* leaves (VISUAL)] (Kondo et al., 2016), in which cotyledon or leaf mesophyll cells are re-programmed to differentiate into a ∼50:50 mix of xylem vessels and sieve elements, sieve element-specific genes can be monitored in unprecedented abundance. Although these trans-differentiated sieve elements are not root protophloem, they do represent a native cell type in which sieve element regulators can be investigated successfully (Breda et al., 2017; Kondo et al., 2016). Moreover, a transcriptomic time series identified *BAM3* as a central node in the early module of the trans-differentiation process (Kondo et al., 2016). Thus, we concluded that VISUAL could be employed to identify CLE26- and CLE45-responsive sieve element-specific genes. To achieve this, we VISUAL-cultured wild-type cotyledons and monitored *BAM3* expression in a parallel cultured transgenic line that carried a *BAM3::GUS* reporter gene. Strong comprehensive *BAM3* expression was observed after 48 h. At this time point, the wild-type sample was split in replicates and treated with control, 100 nM CLE26 or 100 nM CLE45 for 2 h. Total RNA was isolated immediately after from these samples, and cDNA was prepared for high-throughput sequencing. Analysis of the subsequently obtained RNAseq data (Bray et al., 2016; Pimentel et al., 2017) revealed 167 CLE26- and 97 CLE45-responsive genes at a stringent cut off (q value<0.01) (Tables S2,S3), with an overlap of 36 genes between the two sets (Fig. 1A) (Table S4). Interestingly, the top CLE26-responsive gene (in terms of statistical significance) encodes an uncharacterized LRR-RK (At2g23950), which was downregulated (Fig. 1B) (Table S2).

### Forward genetic screening to identify CLE26-resistant mutants

In parallel to the transcriptomic analysis, we performed a forward genetic screen to identify mutants with CLE26-resistant root growth. To achieve this, 870 M2 pools of EMS-mutagenized seeds collected from single M1 plants were grown on media supplemented with 10 nM CLE26 peptide. Of initially 95 lines that displayed at least some CLE26-resistance, 12 could be confirmed in the next generation. Ten lines that were substantially resistant to a higher (100 nM) concentration of CLE26 (Fig. 1C) were subsequently backcrossed to wild type. In the F2 population, segregation of CLE26 resistance indicated recessive inheritance of the phenotype. Fifty to 60 segregating CLE26-resistant and CLE26-sensitive seedlings from one of the lines that displayed the strongest resistance were then pooled, and the genomic DNA of those pools was analyzed by whole-genome sequencing. Comparison of SNP frequencies in the two pools, as described previously (Depuydt et al., 2013; Kang and Hardtke, 2016), pointed to a mutation in At2g23950, the top CLE26-responsive gene in the transcriptomic assay, as causing the CLE26 resistance. This D480N mutation is located in the predicted kinase domain of this LRR-RK (Fig. 1D). We named the gene *CLE-RESISTANT RECEPTOR KINASE* (*CLERK*) after the mutant phenotype. Subsequent analysis of the other lines revealed two more *clerk* alleles: one that carried a premature STOP codon (W65*) and another with a splice acceptor mutation in the eighth intron (Fig. 1D). Finally, a T-DNA insertion allele of *CLERK* obtained from the *Arabidopsis* stock center (line SALK_066568) also displayed strong CLE26 resistance (Fig. 1E). The CLE26 sensitivity in both the original as well as the T-DNA *clerk* mutants could be recovered by introduction of a transgenic *CLERK* wild-type copy (Fig. 1E). In summary, these results prove that *CLERK* is required for CLE26 sensing in the root.

### CLERK is required for full sensing of root-active CLE peptides

**Fig. 2.**
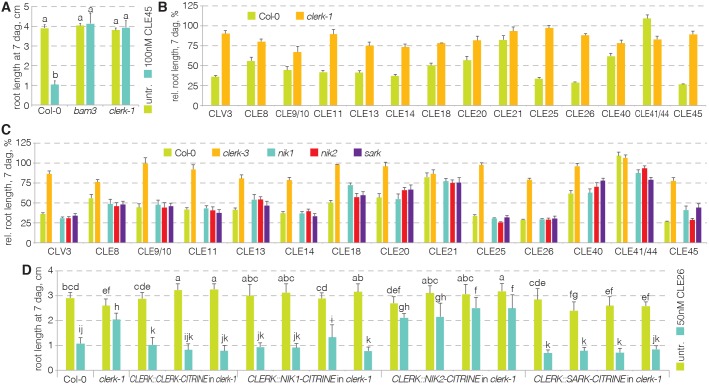
**Resistance of *clerk* mutants to a range of root-active CLE peptides.** (A) Response of *clerk* mutants to CLE45 when compared with Col-0 and *bam3* (ANOVA, *P*<0.001; *n*=16-19). Statistically significant differences are indicated by letters (Tukey test, alpha=0.05). (B) Response of *clerk* mutants to a range of root-active CLE peptides when compared with Col-0 (ANOVA, *P*<0.001; *n*=13-25). (C) Response of indicated mutants to a range of root-active CLE peptides and controls (ANOVA, *P*<0.001; *n*=4-25). Please see Table S1 for statistically significant differences (Tukey test, alpha=0.05). (D) Response of indicated genotypes (several independent transgenic lines per construct) to CLE26 treatment and controls (ANOVA, *P*<0.001; *n*=21-43). Statistically significant differences are indicated by letters (Tukey test, alpha=0.05). See Table S1 for full Tukey test results and ANOVA tables. Error bars represent s.e.m.

Confirmed CLE receptors such as CLV1, BAM3 or PXY are members of the class XI LRR-RKs, the extracellular domains of which are composed of 20-30 LRRs. By contrast, CLERK belongs to the class II LRR-RKs, the extracellular domain of which is typically formed by three or four LRRs (Shiu and Bleecker, 2001). CLERK is therefore in the same, comparatively small, clade that also contains the SERK proteins (Fig. S1A), some of which act as co-receptors for multiple receptors and various ligands (Kemmerling et al., 2007; Meng et al., 2015; Santiago et al., 2016, 2013; Schwessinger and Rathjen, 2015; Torii, 2012). Indeed, in control experiments, we found that *clerk* mutants are also resistant to CLE45 (Fig. 2A). To test whether *CLERK* could further play a role in the sensing of multiple ligands, we assayed the response of *clerk* mutants to various root-active peptides. We observed that *clerk* mutants were not only resistant to CLE26 and CLE45, but also to a range of other CLEs (Fig. 2B). In contrast, no substantial CLE resistance was observed for a loss-of-function mutant in the closest CLERK homolog: the SENESCENCE-ASSOCIATED RECEPTOR-LIKE KINASE (SARK) (Fig. 2C) (Xu et al., 2011). Likewise, available mutants in the closely related NSP-INTERACTING KINASES (NIKs) (Fig. S1A) also behaved like wild type (Fig. 2C). Therefore, the data suggest that *SARK*, *NIK1* and *NIK2* are individually dispensable for CLE sensing in the root, while *CLERK* is required for sensing of multiple CLE peptides. However, when expressed under control of the *CLERK* promoter, both SARK and NIK1 could replace the CLERK protein to sense CLE26 (Fig. 2D). Thus, the CLERK, SARK and NIK1 proteins appear to be functionally equivalent, suggesting sub-functionalization of the corresponding genes.

### CLERK is expressed in early developing protophloem

**Fig. 3.**
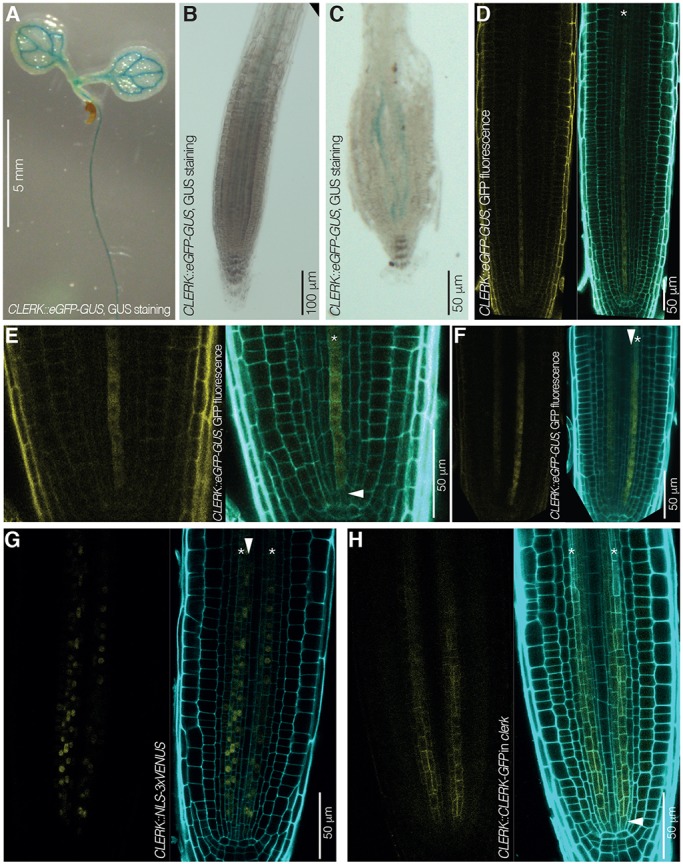
***CLERK* expression pattern in *Arabidopsis* seedlings and roots.** (A-C) *CLERK* expression pattern revealed by GUS staining (blue) in 7-day-old Col-0 seedlings that express an eGFP-GUS fusion under control of the *CLERK* promoter (*CLERK::eGFP-GUS*); light microscopy. (A) Cotyledons and upper part of the root. (B) Higher magnification of the root tip. (C) Higher magnification of the root tip after squashing, revealing expression in two cell files. (D-F) *CLERK* gene expression pattern revealed by GFP fluorescence in root meristems of 7-day-old Col-0 seedlings that carry a *CLERK::eGFP-GUS* transgene; confocal microscopy. Left: GFP fluorescence, yellow. Right: overlay of GFP fluorescence with propidium iodide (PI) cell wall staining (cyan). (D) Root tip overview. Asterisk indicates a protophloem sieve element cell file. (E) Similar to D, higher magnification of the meristem tip, highlighting *CLERK* expression in early protophloem (asterisk) starting next to the protophloem stem cell (arrowhead). (F) Similar to D, alternative perspective angle, highlighting *CLERK* expression in early protophloem sieve elements (asterisk) as well as metaphloem cells (arrowhead). (G) *CLERK* expression visualized by VENUS fluorescence in root meristems of 7-day-old Col-0 seedlings that carry a *CLERK::NLS-3×VENUS* transgene, confocal microscopy. Left: VENUS fluorescence, yellow; Right: overlay of VENUS fluorescence with PI cell wall staining (cyan). Asterisks indicate protophloem sieve element cell files; arrowhead indicates a metaphloem cell file. (H) CLERK protein expression pattern revealed by GFP fluorescence in root meristems of 7-day-old *clerk-3* seedlings that carry a *CLERK::CLERK-GFP* transgene; confocal microscopy. Left: GFP fluorescence, yellow. Right: overlay of GFP fluorescence with PI cell wall staining (cyan). Asterisks indicate protophloem sieve element cell files; arrowhead indicates a phloem stem cell.

To monitor the site of *CLERK* gene expression, we cloned a genomic fragment that encompassed 1.7 kb of promoter sequence and the 5′ UTR in front of an eGFP-GUS fusion reporter gene. In transgenic wild-type plants that were transformed with this construct, GUS reporter activity was evident in the vasculature throughout seedlings (Fig. 3A), but barely visible in the root meristem (Fig. 3B). However, GUS staining was evident in two cell files in the root meristem (Fig. 3C). Matching this observation, the GFP fluorescence in the same lines indicated expression in the root meristem protophloem (Fig. 3D), but was rather faint, suggesting that *CLERK* is expressed at relatively low level. *CLERK* expression was concentrated in the early developing protophloem sieve elements (Fig. 3E). Yet some even weaker expression was also observed in the early metaphloem cell files (Fig. 3F). These observations were confirmed with a *CLERK::NLS-3xVENUS* reporter (Fig. 3G). In comparison with other low abundant protophloem genes, *CLERK* expression was very weak, and faded out as sieve element development progressed towards the differentiation zone. To determine to what degree this gene expression pattern correlates with CLERK protein abundance, the same promoter fragment was isolated together with the entire intron-exon sequence up to the stop codon, and fused in frame to a C-terminal GFP tag. Transgenic *clerk* plants that were transformed with this construct displayed full sensitivity to CLE26 peptide (Fig. 1E), corroborating that the transgene was functional. Expression was observed in the early developing protophloem sieve elements, starting immediately with the phloem stem cell (Fig. 3H; Fig. 5A), and to some degree also in early metaphloem cells. Matching the promoter activity, expression was again comparatively weak (Fig. S1B,C). In summary, the *CLERK* expression pattern is consistent with the central role of the protophloem in the perception of root-active CLE peptides (Hazak et al., 2017).

### CLERK ectodomain does not interact with BAM3 ectodomain

The CLE resistance of *clerk* mutants could be explained through indirect effects, for example on the expression of bona fide CLE receptors, as proposed for *crn* (Hazak et al., 2017). To evaluate this scenario, we focused our efforts on the only identified CLE receptor in the root protophloem so far, the CLE45 receptor BAM3 (Hazak et al., 2017; Kang and Hardtke, 2016). Introduction of a *BAM3::BAM3-CITRINE* transgene into *clerk* mutants did however not reveal any altered BAM3 expression or localization (Fig. S1D). To also test whether CLERK could serve as a co-receptor for BAM3 instead, we obtained purified BAM3 and CLERK ectodomains for biochemical assays. In isothermal titration calorimetry (ITC), no direct binding of the CLERK ectodomain to CLE26 or CLE45 peptide was observed (Fig. S2A,B), unlike high-affinity CLE45 binding to the BAM3 ectodomain (Hazak et al., 2017) or CLE9 binding to BAM1 (Fig. S2C). Moreover, we did not observe CLE45-induced binding of the CLERK and BAM3 ectodomains in gel filtrations (Fig. S3A). Neither did CLERK ectodomain bind to the BAM1 ectodomain or the PXY ectodomain in a CLE9-dependent or CLE41-dependent manner, respectively (Fig. S3B,C). This was in stark contrast to the reported binding of SERK proteins to PXY or receptors of other ligands (Hazak et al., 2017; Hohmann et al., 2017; Zhang et al., 2016a,b). Similar observations were made with ectodomains of several CLERK homologs, including NIK1 (Fig. S3A-F). In summary, these results do not support a direct role for CLERK in BAM3-mediated CLE45 perception.

### CLERK acts genetically independently of CLV2-CRN-mediated CLE signaling

**Fig. 4.**
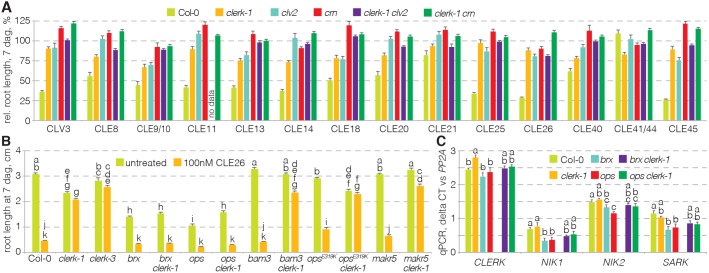
**Epistasis analysis of *clerk* and *clv2-crn*.** (A) Response of indicated genotypes to a range of root-active CLE peptides and controls (ANOVA, *P*<0.001; *n*=5-34). Please see Table S1 for statistically significant differences (Tukey test, alpha=0.05). (B) Response of indicated genotypes to CLE26 peptide (ANOVA, *P*<0.001; *n*=2-25). Statistically significant differences are indicated by letters (Tukey test; see Table S1 for full results and ANOVA table). (C) Expression levels of indicated genes in indicated mutant backgrounds, determined in root tips by qPCR (ANOVA, *P*<0.01 for *CLERK*, *NIK2* and *SARK*, *P*<0.001 for *NIK2*; average of three technical replicates with three biological replicates each). Statistically significant differences are indicated by letters (Tukey test, alpha=0.05). See Table S1 for full Tukey test results and ANOVA tables. Error bars represent s.e.m.

To determine whether CLERK and CLV2-CRN act in concert, we compared the CLE resistance of *clerk* with *clv2* and *crn* mutants. For all three mutants, CLE resistance was roughly similar, although in tendency root growth of *clerk* was slightly more CLE sensitive than of *clv2* or *crn* (Fig. 4A). Epistasis analysis of *clerk clv2* and *clerk crn* double mutants did not reveal any substantial additive or synergistic effects (Fig. 4A), suggesting that CLV2-CRN and CLERK might act in sequence. However, as CLE resistance was already comparatively strong in both *clerk* and *clv2* or *crn* single mutants, corresponding epistasis analysis in double mutants is of limited meaning. An alternative genetic assay to compare the impact of *clerk* and *clv2* or *crn* mutations on protophloem development is to monitor their capacity to suppress the defects in protophloem differentiation mutants such as *brx* or *ops*. Both *clv2* and *crn* second-site mutation have been shown to partially suppress the *brx* phenotype, which likely reflects CLV2-CRN requirement for efficient BAM3 expression in the protophloem (Hazak et al., 2017). By contrast, *clerk brx* double mutants did not display any phenotypic amelioration and, in *clerk ops* double mutants (at best), a partial rescue was observed (Fig. 4B). In summary, the data suggest that the CLV2-CRN and BAM3 pathways on the one side, and the CLERK pathway on the other, are genetically separable.

Evidence for parallel CLE-sensing pathways was also provided by the CLE26 response of the *clerk brx* and *clerk ops* double mutants because, surprisingly, both *clerk brx* and *clerk ops* were CLE26 sensitive (Fig. 4B). This was in contrast to the additive CLE26 resistance phenotype of combinations between *clerk* and specific CLE45-resistant mutants, such as *bam3*, *membrane-associated kinase regulator 5* (*makr5*) (Kang and Hardtke, 2016), or the semi-dominant *ops^E319K^* allele (Rodriguez-Villalon et al., 2014) (Fig. 4B). Thus, in both the *brx* and *ops* null mutant backgrounds, *CLERK* was not required for CLE26 perception. Moreover, consistent with the partial rescue of *brx* by *crn* second site mutation (Hazak et al., 2017), *brx crn* double mutants were still CLE26 sensitive (Fig. S4). These results reiterate the notion that alternative CLE peptide-sensing pathways exist in the root. It appears possible that in *brx* or *ops* mutants, such alternative pathways are upregulated and could compensate for *clerk* loss of function. This might include *CLERK* homologs, and indeed we observed a slight, significant upregulation of *NIK1* and *SARK* expression in both *brx* and *ops* single, as well as the respective *clerk* double mutants (Fig. 4C).

### CLERK prevents premature expression of a sieve element-specific molecular marker

**Fig. 5.**
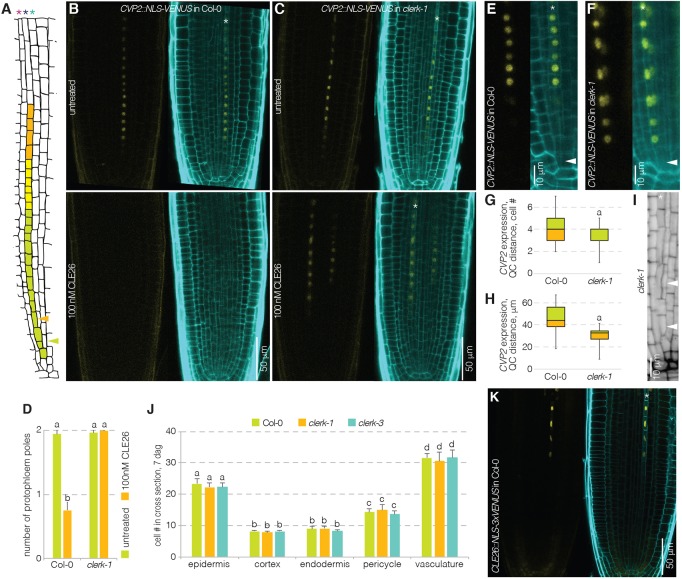
**CLERK prevents premature expression of a protophloem marker.** (A) Schematic presentation of the protophloem sieve element lineage in the *Arabidopsis* root meristem. Red asterisk, pericycle cell file; dark-blue asterisk, protophloem sieve element cell file; light-blue asterisk, incipient metaphloem cell file; arrowheads indicate the two periclinal divisions. (B,C) *CVP2* expression pattern in response to mock or CLE26 treatment, revealed by VENUS fluorescence in root meristems of 5-day-old Col-0 or *clerk* seedlings that carry a *CVP2::NLS-VENUS* transgene; confocal microscopy. Left: VENUS fluorescence, yellow. Right: overlay of VENUS fluorescence with PI cell wall staining (cyan). Asterisk indicates protophloem sieve element cell file. (D) Quantification of protophloem strand presence/absence in root meristems of a Col-0 wild type or *clerk* mutant upon CLE26 treatment. Statistically significant differences when compared with mock control are indicated by letters (Fisher's *F*-test, *P*<0.001; *n*=18-30). (E,F) Higher magnifications of *CVP2* expression in early protophloem of Col-0 or *clerk* (untreated seedlings). (G) Distance between first detectable *CVP2* expression and the quiescent center (QC) in Col-0 wild type and *clerk* mutants, expressed as cell number. Statistically significant difference when compared with Col-0 wild type is indicated (Student's *t*-test, *P*<0.05; *n*=13-19). (H) Distance between first detectable *CVP2* expression and the quiescent center (QC) in Col-0 wild type and *clerk* mutants, expressed as absolute distance. Statistically significant difference as compared to Col-0 wild type is indicated (Student's *t*-test, *P*<0.001; *n*=13-19). In G,H, box and whiskers represent maximum, 3rd quartile, 2nd quartile and minimum values. (I) Higher magnification of the early protophloem sieve element lineage in a *clerk-1* mutant; mPS-PI staining (gray); confocal microscopy. Arrowheads indicate the two periclinal divisions (compare with A). (J) Quantification of cell file number in Col-0 wild type or *clerk* mutants, counted at the level where protoxylem has differentiated. None of the differences within cell types was statistically significant (*n*=8 or 9) (Tukey test; letters indicate statistical differences; see Table S1 for full results and ANOVA table). (K) *CLE26* expression pattern as revealed by VENUS fluorescence in root meristems of 7-day-old Col-0 seedlings that carry a *CLE26::NLS-3×VENUS* transgene; confocal microscopy. Left: VENUS fluorescence, yellow; Right: overlay of VENUS fluorescence with PI cell wall staining (cyan). Asterisk indicates protophloem sieve element cell file. Error bars represent s.e.m.

In the *A. thaliana* root meristem, protophloem sieve elements are produced by a highly stereotypic developmental pattern (Fig. 5A). Following anticlinal division of a single stem cell, the sieve element-procambium precursor daughter cell switches division plane to produce an inner procambial cell and an outer sieve element precursor cell by periclinal division (Bonke et al., 2003; Rodriguez-Villalon et al., 2014, 2015). The sieve element precursor cell eventually performs a second periclinal division, which gives rise to an inner incipient metaphloem precursor cell and an outer protophloem precursor cell. The protophloem precursor cell then undergoes repeated anticlinal divisions before the onset of differentiation. The *COTYLEDON VASCULAR PATTERN 2* (*CVP2*) gene is a specific marker of this process, which is expressed after the first periclinal division, and stays on exclusively in the developing protophloem after the second periclinal division (Rodriguez-Villalon et al., 2014, 2015). Application of root-active CLE peptides suppresses sieve element differentiation and thus also *CVP2* expression (Hazak et al., 2017; Rodriguez-Villalon et al., 2015). In line with their CLE resistance, the roots of *clerk* mutants maintained protophloem development in the presence of CLE26 peptide (Fig. 5B-D). Moreover, compared with wild type, expression of the *CVP2::NLS-VENUS* marker appeared markedly earlier in *clerk* mutant background, frequently already in the first sieve element-procambium precursor that directly neighbored the stem cell (Fig. 5E-H). Because both periclinal divisions of the phloem lineage were maintained (Fig. 5I) and no difference in formative divisions was observed (Fig. 5J), this indicates that the onset of protophloem differentiation could be premature in *clerk* mutants. The consequent notion that *CLERK* might restrict the transition to sieve element differentiation in early protophloem matches with its expression pattern. Moreover, along the developing sieve element strand, the *CLERK* and *CLE26* expression patterns were largely complementary, with a short overlap (Fig. 5A,K). Given the CLE26-responsiveness of *CLERK* in the VISUAL assay, this could reflect CLE26-mediated *CLERK* downregulation during progression of sieve element development. However, an equally strong effect was not observed in the root meristem (Fig. S1E).

## DISCUSSION

The *Arabidopsis* genome contains a large number of orphan RKs and peptide ligands (Liu et al., 2017; Shiu and Bleecker, 2001). Even within the smaller group of CLE peptides and receptors, relatively few genetically coherent and biochemically confirmed ligand-receptor pairs are known to date (Brandt and Hothorn, 2016; Endo et al., 2013; Etchells and Turner, 2010; Hazak et al., 2017; Hazak and Hardtke, 2016; Hirakawa et al., 2008; Hohmann et al., 2017; Ogawa et al., 2008; Rojo et al., 2002; Shinohara and Matsubayashi, 2015). However, because of the substantial structural similarity within subgroups of RK proteins as well as CLE peptides, it is conceivable that there is considerable redundancy. Indeed, genetic cross-complementation as well as promiscuous biochemical interactions have been observed, suggesting that CLE peptide-receptor pairs are to some degree interchangeable (Deyoung and Clark, 2008; Endo et al., 2013; Hohmann et al., 2017; Nimchuk, 2017; Nimchuk et al., 2015; Shinohara et al., 2012; Zhang et al., 2016a). Yet beyond biochemical possibilities, *in planta* the affinities of individual receptor-ligand pairs come into play, as well as limitations on the formation of possible receptor-ligand complexes that are imposed by specific expression patterns and levels. Our observation that some homologs can replace *CLERK* in CLE26 sensing in *A. thaliana* root protophloem at the protein level, but not at the genetic level, reiterates this notion.

The topology of the ectodomain of CLERK and its phylogenetic position in a clade together with SERKs suggests that CLERK likely acts as a co-receptor, rather than a receptor, for CLE peptide signaling. This idea is also in line with its promiscuity, a feature that is shared by SERK proteins, which not only signal in conjunction with different receptors, but even with different ligands (Brandt and Hothorn, 2016). Thus, unless its impact on CLE sensing is indirect, CLERK might serve as a co-receptor for various CLE receptors in the root. Our experimental evidence suggests that this does not include the CLE45 receptor BAM3, although it remains formally possible that CLERK acts in concert with BAM3 but requires additional, so far unknown, factors. However, any such interaction would have to occur in a biochemical manner that is fundamentally different from the described interactions, for which receptors, ligands and SERKs are sufficient. Alternatively, redundant CLE45 receptors might exist in the root. Conversely, the CLE26 sensitivity of *clerk brx* and *clerk ops* double mutants suggests that *CLERK* can be replaced by redundant pathways or RKs, for example other members of the class II LRR-RKs. Even though neither SARK nor NIK1 appear to have an individual role in CLE sensing in the root, they, or other clade members, might replace *CLERK* in a particular genetic scenario; e.g. if crossregulation between *CLERK* and other clade members exists, i.e. similar to the precedence set by cross-regulation between CLV1 and the redundant BAM receptors in the shoot meristem (Nimchuk, 2017), the absence of *CLERK* and *BRX* (or *OPS*) might lead to de-repression of a redundant co-receptor in the *CLERK* expression domain. Indeed, the observation that, similar to *CLERK*, both *SARK* and *NIK2* were downregulated by CLE26 treatment in the transcriptomic analysis (see Table S2), and that *NIK1* and *SARK* are upregulated in the absence of *BRX* or *OPS*, points to possible crossregulation between class II LRR-RKs. Comprehensive investigation of the expression patterns of all clade members in wild-type and pertinent mutant backgrounds is thus warranted and could provide valuable guidance in future analyses. Finally, while this manuscript was under revision, redundancy between the receptors described here has indeed been reported in shoot development (Hu et al., 2018). This study also implicates additional players in the pathway, for which a role has also been described in the root (Racolta et al., 2018). Collectively, these and our results highlight not only the intricate complexity of CLE-sensing pathways, but also their context-specific action.

The incapacity of *clerk* loss-of-function second site mutation to suppress the *brx* phenotype (unlike second site *bam3* or *clv2-crn* loss of function) is also of interest with respect to the role of CLE peptide signaling in the spatiotemporal progression of protophloem sieve element differentiation. Resistance of *clerk* mutants against root-active CLE peptides as well as *CLERK* expression in early protophloem reiterate the key role of the protophloem in CLE sensing in the root (Hazak et al., 2017). Yet not all CLE peptides have an equally strong effect on root growth, which correlates with their impact on sieve element differentiation, as judged from suppression of the *CVP2::NLS-VENUS* marker to varying degrees (Hazak et al., 2017). Given the opposite phenotype, the premature expression of *CVP2* in *clerk* mutants, this might mean that many externally applied root-active CLEs act primarily during the early stages of protophloem formation, on the principal *CLERK* expression domain. The fact that *crn* second site loss-of-function rescues *brx* might thus reflect the specific impact of CLV2-CRN on stable BAM3 expression along the entire developing protophloem (Hazak et al., 2017). That is, unlike most other root-active CLEs, CLE45 (which is also the strongest root-active CLE peptide) could act on the entire developing protophloem through its receptor BAM3. By contrast, *CLERK* loss of function apparently mainly affects specification timing of early protophloem. This might be less important in the context of *brx* or *ops* mutations, which mainly affect the differentiation process in later protophloem. Our findings therefore also suggest an intricate interplay between CLE-sensing pathways along the spatiotemporal gradient of protophloem formation. Our discovery of CLERK as a crucial component of CLE sensing in the early protophloem can serve as a starting point in dissecting the different stages of this process, by providing a sensitized background for future screens.

## MATERIALS AND METHODS

### Plant growth conditions

*Arabidopsis thaliana* Columbia-0 (Col-0) was used as the wild type for phenotypic analyses and is the background of all mutants investigated: *ops-2* (SALK_139316); *brx-2* (Rodrigues et al., 2009); *bam3-2* (SALK_044433) (DeYoung et al., 2006); *clerk-3* (SALK_066568); *ops^E319K^* (Rodriguez-Villalon et al., 2014); *makr5* (Kang and Hardtke, 2016); *nik1* (SALK_017538C); *nik2* (SALK_044363C); and *sark* (SALK_111290), *clv2/rlp10-1* (GK-686A09) and *crn-10* (Hazak et al., 2017). Seeds were surface sterilized, germinated and grown vertically under continuous light at 22°C on 0.5× Murashige and Skoog media supplemented with 1% agar and with or without 0.3% sucrose.

### Physiological assays

CLE peptides were obtained from a commercial supplier (Genscript), synthesized at >80% purity, diluted in water and used at final concentration as indicated. For root length measurements, plates were scanned at 600 dpi resolution and seedling root length was determined using the Simple Neurite Tracer plug-in (Longair et al., 2011) for Fiji software (Schindelin et al., 2012).

### Cloning

PCR products were amplified with high fidelity polymerase (Kapa Hifi, Roche Diagnostics, or Q5, NEB) from genomic DNA and successively cloned into pDONR221 or pDONR207 by BP reaction, then sequenced and transferred into pDEST vector by LR reaction using Gateway cloning strategies (Invitrogen). The *CLERK* promoter including the 5′UTR (1729 bp upstream of the ATG start codon) was amplified (oligonucleotides 5′-GGG GAC AAG TTT GTA CAA AAA AGC AGG CTT AAC CGG GCC ATT AGT GGG AAC GAG-3′ and 5′-GGG GAC CAC TTT GTA CAA GAA AGC TGG GTA GAA AAG CTT TAG ATT AGA TTA CCA G-3′) and cloned into pDONR221, then recombined into pBGWFS7,0 destination vector (Karimi et al., 2002). The same promoter followed by the genomic *CLERK* transcript region was cloned without the stop codon into pDONR207 (oligonucleotides 5′-GGG GAC AAG TTT GTA CAA AAA AGC AGG CTT AAC CGG GCC ATT AGT GGG AAC GAG-3′ and 5′-GGG GAC CAC TTT GTA CAA GAA AGC TGG GTA CCT TGG ACC AGA TAG TTC CAT GGC-3′) and recombined into pGWB4 destination vector for translational fusion with GFP. The *BAM3* promoter has been previously described (Rodriguez-Villalon et al., 2014). For the *BAM3::CLERK-CITRINE* construct, the genomic *CLERK* transcript region was cloned into pDONR221 without stop codon (5′-GGG ACA AGT TTG TAC AAA GCA GGC TGG ATG GTG ATG AAG TTA ATA AC-3′ and 5′-GGG GAC CAC TTT GTA CAA GAA AGC TGG GTA CCT TGG ACC AGA TAG TTC CAT GGC-3′), recombined and fused to CITRINE in the pH7m34GW destination vector. The *CLE26* promoter (Rodriguez-Villalon et al., 2015) was amplified and cloned into pCAMBIA 1305.1 using *Sal*I-*Xba*I restriction sites to drive expression of an NLS-3×-mVENUS cassette. The final constructs were introduced into *A. thaliana* by standard *Agrobacterium tumefaciens*-mediated (strain GV3101 with pMP90 helper plasmid) floral dipping transformation. Transgenic plants were selected for their hygromycin resistance in tissue culture (35 mg/ml hygromycin), and single insertion lines were studied.

### RNA sequencing

Vascular tissue trans-differentiation was induced in cotyledons over 2 days as described previously (Kondo et al., 2016). Samples were then treated with 100 nM of CLE26 or CLE45 peptide for 2 h. Total RNA of two biological replicates was then extracted from 100 mg of fresh material, using RNeasy Plant mini kits (Qiagen). Truseq Stranded mRNA kits (Illumina) were used to prepare cDNA libraries, which were sequenced on an Illumina Hiseq 2500 instrument. Read counts and differential expression were determined with Kallisto (Bray et al., 2016) and Sleuth (Pimentel et al., 2017) software, using the TAIR10 reference transcriptome. The raw data have been deposited in the NCBI Sequence Read Archive (www.ncbi.nlm.nih.gov/sra/) under accession SRP126390.

### *clerk* mutant isolation

M2 mutagenized seeds (Kalmbach et al., 2017) were screened in tissue culture on media complemented with 10 nM CLE26 peptide. Seedlings with CLE26-resistant root growth were transferred to soil and the phenotype was confirmed in the M3 generation before backcrossing to Col-0 wild type. The causative mutation for CLE26 resistance was then determined by whole-genome sequencing of bulked segregants as described previously (Kang and Hardtke, 2016).

### Genotyping

*clerk-1* was genotyped using a dCAPS strategy. A 200 bp fragment was amplified using oligonucleotides 5′-GAT GTC AAG GCA AAC ATT C-3′and 5′-AAA AGT ATA CCG AAC CCA AAG ATA T-3′. The PCR product was then digested with *Eco*RV restriction enzyme, which cut the Col-0 wild-type fragment into two pieces of 174 bp and 26 bp, but not the *clerk1* fragment. For genotyping of T-DNA insertion lines, the following oligonucleotides were used to amplify the wild-type alleles: *CLERK*, 5′-ATC CTT GTA GCT GGA CTA TG-3′ and 5′-GGA ACA GGA CCT CTG AGA TTG-3′; *NIK1*, 5′-GAC AAA AAC ATG ACA GGG TGG-3′ and 5′-CAT TGT TTT CCT TGC TTG CTC-3′; *NIK2*, 5′-CCA AAG AAA ACC AAA GCC-3′ and 5′-AGA GAA GCT CCA AGC CAA AAC-3′; *SARK*, 5′-AAC AAG AGG AAG GGC TTC AAG-3′ and 5′-ATG TGA ATG GTT ATG CGA AGC-3′; *BAM3*, 5′-CTG CAA CTT CTC CGT TTG-3′ and 5′-GAT TCC TTC GAA ACT CGG ATC-3′. To detect the T-DNA insertion alleles, the above oligonucleotides were combined with oligonucleotide LbB1.3 (5′-ATT TTG CCG ATT TCG GAA C-3′). *CLV2* was genotyped with 5′-GTC TAG CTT GTC AGA ATC C-3′ and 5′-TTA AGA ACC AAT GG-3′ for the wild-type allele, and in combination with 5′-CCC ATT TGG ACG TGA ATG TAG ACA C-3′ for the *rlp-10* mutant allele of *clv2*. *ops-2* was genotyped as described previously (Anne et al., 2015). The *crn-10* allele was detected by amplification of a 330 bp PCR product using oligonucleotides 5′-GTA GAA GCA ATG AAG CAA AGA AGG TG-3′ and 5′-GTT GAA GTT GTG GAT AAG TG-3′, and restriction digest with *Hph*I, cutting the *crn-10* allele fragment (but not the wild-type allele fragment) into 289 bp and 41 bp. The *makr5* allele was detected by amplification of a 200 bp PCR product using oligonucleotides 5′-GAA GCT CTT ACC TTT ATG AAA TAC TA-3′ and 5′-GTT GTT TCG AGT CTC TG-3′, and restriction digest with *Spe*I, cutting the wild-type allele fragment (but not the *makr5* allele fragment) into 180 bp and 20 bp. *BRX* was genotyped using 5′-GTC AGT GTT TGC TTC CTC TCT ATG-3′ and 5′-TAT TTC CTT GTC TAG GTA AGA ATC C-3′ for the wild-type allele, and in combination with 5′-TGA TCC ATG TAG ATT TCC CGG ACA TGA A-3′ for the *brx-2* allele. The *ops^E319K^* allele was detected by amplification of a 145 bp PCR product using oligonucleotides 5′-CTT CAG AAA TGG AGG CAG AAT-3′ and 5′-CAT ATC CGT AAT CAG CAA GCT-3′, and restriction digest with *Hin*dIII, cutting the *ops^E319K^* allele fragment (but not the wild-type allele fragment) into 125 bp and 20 bp.

### Protein expression, isolation and analytical size exclusion chromatography

Isolation of recombinant proteins and analytical gel filtrations were carried out as described previously (Hazak et al., 2017) with small adjustments. The coding sequences for BAM1-ECD (amino acids 20-637), BAM3-ECD (30-651), PXY-ECD (30-647), NIK1-ECD (32-248), NIK2-ECD (33-248), NIK3-ECD (26-238), CLERK-ECD (28-236) and At5G63710-ECD (51-224) were amplified from *Arabidopsis thaliana* cDNA using PfuX7 polymerase. All ECD-coding sequences were then cloned into a modified pFAST-BAC1 vector (Geneva Biotech) by GIBSON cloning, resulting in the addition of the azurocidin signal peptide sequence to the 5′-end, and of a 2×-STREP-9×HIS tag to the 3′-end of the ECD sequences. These plasmids, after being confirmed by sequencing, were transformed into *Escherichia coli* DH10MultiBac (Geneva Biotech) followed by the extraction of the resulting bacmids. Transfection of the bacmids into *Spodoptera frugiperda* Sf9 cells with Profectin (AB Vector) was carried out to produce and amplify the respective virus. Recombinant secreted expression of the proteins was carried out by addition of virus to *Trichoplusia ni* Tnao38 cells to reach a viral multiplicity of 1 and incubation of the cells for 3 days. The ECDs were then isolated by removing the cells by centrifugation and subjecting the supernatant to Ni^2+^-affinity chromatography (HisTrap Excel; GE Healthcare). The purity of the proteins was further increased by a StrepII-affinity purification step (Strep-Tactin XT Superflow high capacity, IBA). After dialyzing the proteins towards 20 mM citrate (pH 5.0) and 150 mM NaCl, the proteins were further purified by means of preparative size exclusion chromatography with a HiLoad 26/600 Superdex 200 pg (BAM1-ECD, BAM3-ECD and PXY-ECD; GE Healthcare) or a Superdex 200 Increase 10/300 GL (NIK1-ECD, NIK2-ECD, NIK3-ECD, CLERK-ECD and At5G63710-ECD; GE Healthcare) column equilibrated with 20 mM citrate (pH 5.0) and 150 mM NaCl. Monomeric peak fractions were pooled and concentrated using Amicon Ultra concentrators (Millipore). Concentrations of the pure proteins were assessed by measuring their absorbance at 280 nM (NanoDrop Lite; Thermo Scientific) and subsequent correction with their respective extinction coefficient. For analytical size exclusion chromatographies, 100 µg of each NIK-ECD, CLERK-ECD or At5G63710-ECD were mixed with equimolar amounts of either BAM1-ECD, BAM3-ECD or PXY-ECD, and 50 µM of CLE9 [RLV(HYP)SG(HYP)NPLHN; HYP indicates hydroxy-proline], CLE41 (HEVPSGPNPISN) or CLE45 (RRVRRGSDPIHN) peptide (PSL GmbH), respectively. The mixtures, as indicated in the figures, were subjected to analytical size exclusion chromatography on a Superdex 200 Increase 10/300 GL column (GE Healthcare) with 20 mM citrate (pH 5.0) and 150 mM NaCl as running buffer. Indicated fractions were run on Bis-Tris acrylamide gels and then Coomassie Blue stained.

### Isothermal titration calorimetry

Isothermal titration calorimetry experiments were carried out using the Nano ITC (1 ml standard cell; 250 µl syringe; TA Instruments) with the size exclusion buffer [20 mM citrate (pH 5.0) and 150 mM NaCl] at 25°C. CLE9, CLE26 (RKVPRGPDPIHN) and CLE45 were weighed and dissolved in 20 mM citrate (pH 5.0) and 150 mM NaCl. CLE26 or CLE45 (400 µM) were titrated into 30 µM CLERK-ECD and 80 µM CLE9 was titrated into 13.8 µM BAM1-ECD. Each 15 µl were injected into the cell 17 (CLERK versus CLE26) or 16 (CLERK versus CLE45) times every 150 s. For BAM1 versus CLE9, the injection volume was 10 µl for each of the 24 injections with an injection interval of 150 s. The measurements were corrected by subtracting the heat rates resulting from injecting the peptides with the same concentrations into a cell containing 20 mM citrate (pH 5.0) and 150 mM NaCl. Curve fitting and data analyses were performed with the manufacturer's software (NanoAnalyze, version 3.5).

### Expression analysis by qPCR

RNA from 0.5 cm long root tips of 7-day-old seedlings was isolated using RNeasy Plant mini kits (Qiagen), and cDNA was produced by reverse transcription (Invitrogen). Real-time quantitative PCR was performed in triplicate on three biological repeats using MESA Blue qPCR MasterMix Plus for SYBR Assay Low Rox (Eurogentec) according to the manufacturer's instructions. The PP2A-A3 (At1g13320) gene was used as a reference (primers 5′-GCA ATC TCT CAT TCC GAT AGT C-3′ and 5′-ATA CCG AAC ATC AAC ATC TGG-3′). Following primer efficiency tests, primers 5′-TTA ACA AAT CTT CGG ATT GTG C-3′ and 5′-AAG AGT CTC AAG CCT CGT AAG C-3′ were chosen to monitor *NIK1* expression; primers 5′-CAG ACA ACC TCG TAA TTG GCT TA-3′ and 5′-GAC CCA GAT AAA GTT CCT GAA AGA-3′ to monitor *CLERK* expression; primers 5′-TGT CGG AGA TTT CGG GTT GG-3′ and 5′-GAC CCA CTG TTC CTC TCA CG-3′ to monitor *NIK2* expression; and primers 5′-TTA CCC TTA CAT GCC TAA TGG AA-3′ and 5′-AAC AAA CCT CTC GCT GCA C-3′ to monitor *SARK* expression.

### Statistical analysis

ANOVA analyses were carried out using RStudio software (www.rstudio.com/), Tukey tests were performed using the *agricolae* package (cran.r-project.org/web/packages/agricolae/index.html) with a confidence level alpha of 0.05.

### GUS staining

For GUS staining, samples were fixed in 90% (v/v) acetone for 1 h at −20°C. Samples were then incubated in 5 mM potassium ferricyanide and 5 mM potassium ferrocyanide in 100 mM sodium phosphate buffer (pH 7.2) containing X-gluc at 37°C for 48 h in the dark. Samples were then cleared with ethanol washing steps (30% to 90%) and rehydrated, mounted on slides with water and observed under a light microscope.

## Supplementary Material

Supplementary information
