# Supplementary material for: CLERK is a novel receptor kinase required for sensing of root-active CLE peptides in Arabidopsis
Source: Development. 2018 May 22;145(10):dev162354. doi: 10.1242/dev.162354 (PMC6001375; doi:10.1242/dev.162354)
Supplement: Supplementary information [file develop-145-162354-s1.pdf]

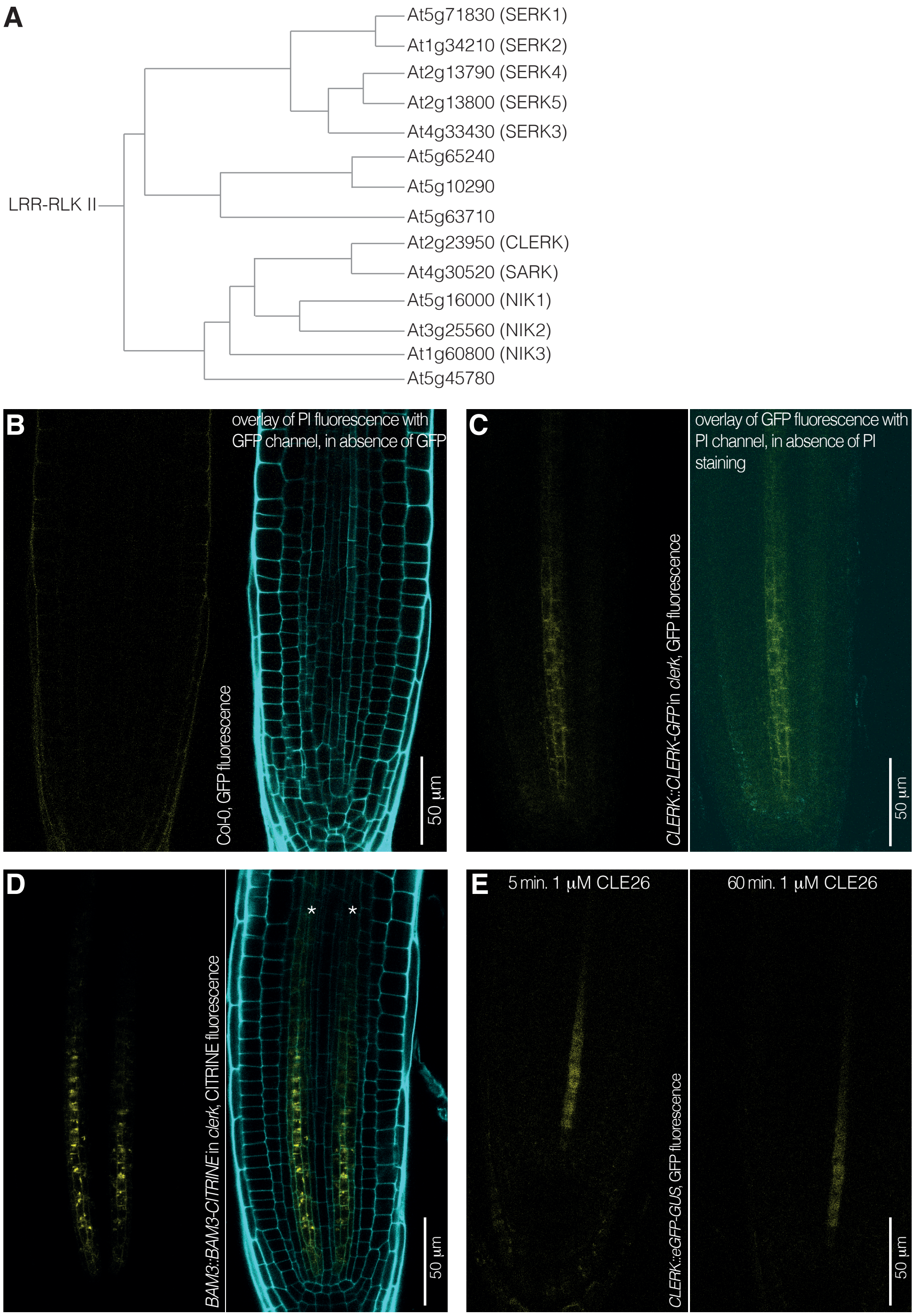

Figure S1 – Supplemental figure. (A) Phylogenetic tree of the class II LRR-RKs of Arabidopsis. (B) Col-0 wild type control root meristem, 5 days old, confocal microscopy. Left: GFP channel, yellow, note some background on the root edges; Right: Overlay of GFP channel with PI cell wall staining (cyan). (C) CLERK-GFP fusion protein expression pattern, confocal microscopy, imaged without PI cell wall staining. Left: GFP fluorescence, yellow; Right: Overlay of GFP fluorescence with PI channel, note increased background. (D) BAM3-CITRINE fusion protein expression pattern in a clerk mutant root meristem, confocal microscopy. Left: CITRINE fluorescence, yellow; Right: Overlay of CITRINE fluorescence with PI cell wall staining (cyan). Asterisks indicate protophloem sieve element cell files. (E) CLERK gene expression in response to CLE26 treatment, as monitored by GFP fluorescence in the root meristem of a Col-0 seedling that carried a CLERK::eGFP-GUS transgene, confocal microscopy. The same root is shown at the beginning and end of a time course. GFP fluorescence, yellow;

Development • Supplementary information

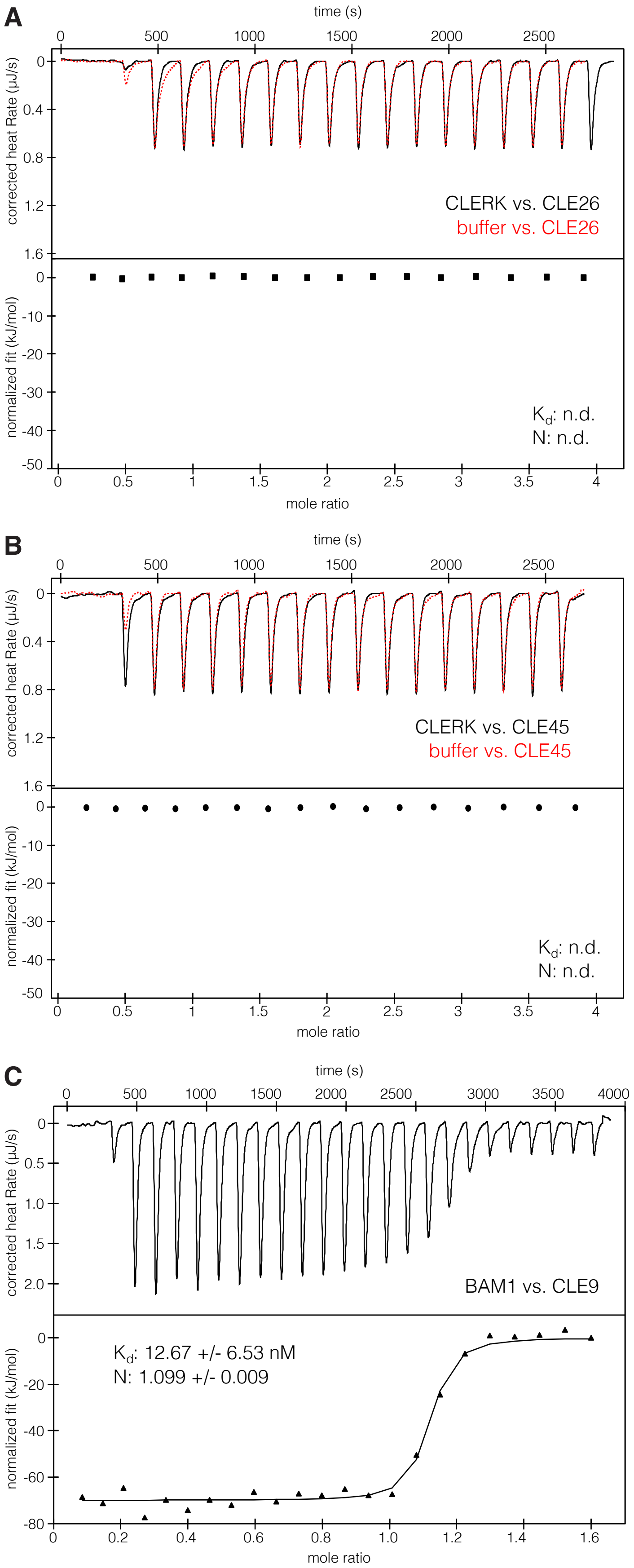

Figure S2 – Supplemental figure. (A-C) Isothermal titration calorimetry (ITC) of purified CLERK or BAM1 ectodomain versus CLE26, CLE45 or CLE9 peptide, respectively (black), as compared to buffer controls (red). N: stoichiometry, Kd dissociation constant. Shown are experimental values ± fitting errors (95% confidence interval).

Development • Supplementary information

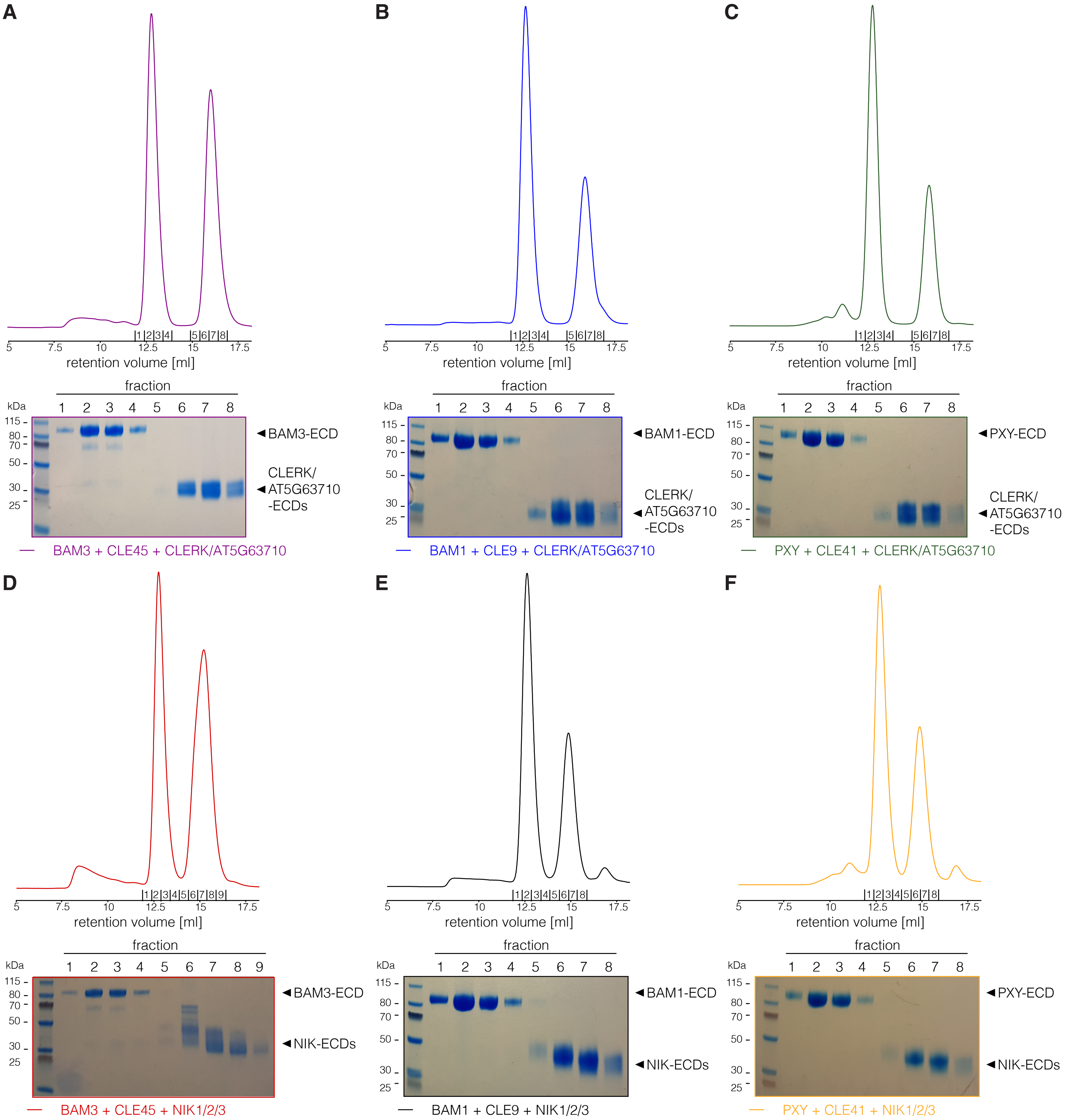

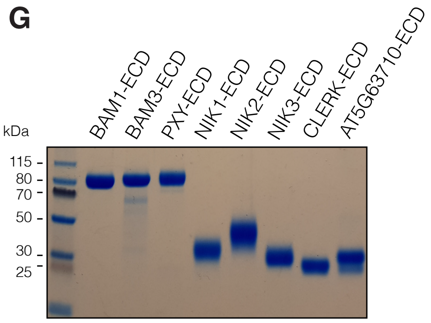

Figure S3 – Supplemental figure. (A-F) Analytical size-exclusion chromatography. The BAM1, BAM3 and PXY LRR ectodomains elute as monomers when run in combination with ectodomains of CLERK and it homologs, and in the presence of CLE peptides. SDS–PAGE gels of the corresponding peak fractions are shown alongside.
(G) SDS-PAGE gels of the purified ectodomains used as input in A-F.

Development • Supplementary information

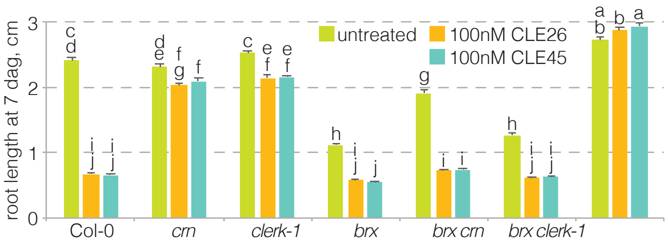

Figure S4 – Supplemental figure. Response of indicated genotypes to CLE26 or CLE45 peptide (ANOVA, $p < 0.001$; n=24-44). Statistically significant differences are indicated (Tukey test; see Supplemental Data 1 for full results and ANOVA table).

Table S1 – ANOVA tables and Tukey test details (Excel file).

Click here to Download Table S1

Table S2 – Differential gene expression upon CLE26 treatment in the VISUAL assay (mock over CLE26 treatment) as determined by RNAseq (Excel file).

Click here to Download Table S2

Table S3 – Differential gene expression upon CLE45 treatment in the VISUAL assay (mock over CLE45 treatment) as determined by RNAseq (Excel file).

Click here to Download Table S3

Table S4 – Overlap of CLE26- and CLE45-responsive genes in the VISUAL assay, with $q < 0.01$ (Excel file).

Click here to Download Table S4

Development • Supplementary information
